# Advancing knowledge of the plant nuclear periphery and its application for crop science

**DOI:** 10.1080/19491034.2020.1838697

**Published:** 2020-12-09

**Authors:** David E. Evans, Sarah Mermet, Christophe Tatout

**Affiliations:** a Department of Biological and Medical Sciences, Oxford Brookes University, Oxford, UK; b GReD, CNRS, INSERM, Université Clermont Auvergne, Clermont-Ferrand, France

**Keywords:** LINC Complex, sun domain, KASH domain, nucleoskeleton, crop improvement, rice, maize, Medicago

## Abstract

In this review, we explore recent advances in knowledge of the structure and dynamics of the plant nuclear envelope. As a paradigm, we focused our attention on the Linker of Nucleoskeleton and Cytoskeleton (LINC) complex, a structurally conserved bridging complex comprising SUN domain proteins in the inner nuclear membrane and KASH domain proteins in the outer nuclear membrane. Studies have revealed that this bridging complex has multiple functions with structural roles in positioning the nucleus within the cell, conveying signals across the membrane and organizing chromatin in the 3D nuclear space with impact on gene transcription. We also provide an up-to-date survey in nuclear dynamics research achieved so far in the model plant *Arabidopsis thaliana* that highlights its potential impact on several key plant functions such as growth, seed maturation and germination, reproduction and response to biotic and abiotic stress. Finally, we bring evidences that most of the constituents of the LINC Complex and associated components are, with some specificities, conserved in monocot and dicot crop species and are displaying very similar functions to those described for *Arabidopsis*. This leads us to suggest that a better knowledge of this system and a better account of its potential applications will in the future enhance the resilience and productivity of crop plants.

## Introduction to the plant nuclear periphery

The aim of this review is to explore the application of recent advances in knowledge of the structure and dynamics of the plant nucleus and its future application in enhancing the resilience and productivity of crop plants. The nucleus is a highly complex compartment and its functions depend on its ordered and dynamic structure. In a living cell, the nucleus is capable of movement, changes in shape and volume, and reorganizing chromatin to alter the position of genetic material. It responds to physical and environmental stimuli. It is organized spatially by the presence of proteins that form a scaffold-like structure supporting, moving and linking the nucleus, its membranes and contents.

While the principles of the structural organization of the nucleus are universal to all eukaryotes studied, studies have revealed that plants have a unique combination of proteins achieving these functions [1–3]. These components include functional homologues of the nucleoskeleton and of proteins that are intrinsic to the inner and outer nuclear membrane (INM and ONM) and that link to the cytoskeleton. While showing structural conservation, the nuclear pore complex (NPC) also has unique features [4] although it will not be the focus of this review. Here we will review knowledge of the plant nuclear periphery focusing on three main components. First, the proteins of the ONM linking with the cytoskeleton, organelles and the plasma membrane to provide a physical signaling pathway and to position and move the nucleus. Second the proteins of the INM, acting as an intermediary in the transmission of signals from outside the nucleus into the nucleus and chromatin through their connection with the nucleoskeleton. Finally, the nucleoskeleton, connected to chromatin to achieve, inter alia, chromosome positioning and 3D structure of the genome allowing changes in expression of the genome.

## The plant Linker of Nucleoskeleton and Cytoskeleton complex

At the center of this interlinked chain of proteins is the Linker of Nucleoskeleton and Cytoskeleton (LINC) complex. It is structurally conserved in all eukaryotes, made up of a protein bridge spanning the INM and ONM across the nuclear periplasm with anchors to link to the structural proteins of the nucleoplasm and cytoplasm. A small family of INM proteins, Sad1/UNC84 homology (SUN) domain proteins are highly conserved; two, SUN1 and SUN2 are present in plants. These have a C-terminal SUN domain (Cter-SUN) in the periplasm which interacts with a second family of proteins located in the ONM, the Klarsicht/ANC-1/Syne Homology (KASH) domain proteins. In addition, between one and three mid-SUN proteins are present, having the SUN domain in a central position. While the Cter-SUN are highly enriched in the INM, a substantial fraction of mid-SUNs is also ER localized [5,6].

KASH domain proteins are structurally conserved but difficult to identify in plants due to absence of sequence conservation. The SUN-binding KASH domain is located in the nuclear periplasm and together with the SUN domain they form the bridging complex. KASH domain proteins are very variable in structure and function [7]. The first plant KASH proteins identified were WPP-domain interacting proteins (WIPs) that anchor Ran GTPase activating protein (RanGAP) to the NE [7], a key mechanism required for nucleocytoplasmic transport through the NPC. WIPs have a typical KASH family structure, including, a transmembrane domain and a short SUN-domain interacting sequence of the three amino acids VPT [2,8]. They also have a cytoplasmic coiled-coil domain. Anchorage of RanGAP to the envelope involves SUN domain proteins, two KASH domain proteins, WIPs (WIP1 and WIP2) and further associated proteins, the WPP domain-interacting tail anchored proteins, WIT1 and WIT2. It may also include further WPP proteins [7].

The second family of plant KASH domain proteins described were the SINEs (SUN-interacting NE proteins) [1]. *SINE1* and *SINE2* of *Arabidopsis thaliana* (*A. thaliana*), are both expressed in roots, they show differential expression in leaves [9], with *SINE1* expressed in guard cells and their progenitors, while *SINE2* is expressed in trichomes, epidermal and mesophyll cells, and only weakly in mature guard cells [9]. SINE1 and SINE2 have an N-terminal with homology to armadillo (ARM) repeat domains, which are known to bind actin and act as protein protein interaction domains [10]. Co-localization with actin has been verified for SINE1 but not for SINE2. While SINE1 has a function in guard cell movement [9,11], SINE2 has been shown to be involved in immunity to a plant pathogen [9]. SINEs 3–4 have short, unique cytoplasmic domains and their function is yet to be fully described. In addition, a further dicot KASH domain protein was described. The Toll Interleukin Receptor (TIR) domain KASH protein (TIK) was identified during studies of mid-SUN interactors [6]. AtTIK has a TIR domain and a C-terminal TM domain upstream of a PPPS motif, a characteristic signature of the KASH domain and appears to be present only in a few Brassicaceae species (*A. thaliana, A. lyrata*, …) [6]. Most recently, a novel family of KASH domain proteins has been described in the graminae. Named Maize LINC KASH Grass-specific (MLKG) [12], they are described in detail for maize (below).

## The higher plant nuclear lamina

The protein composition of the plant nuclear lamina is actively being researched. The lamina in animals, classically described as a layer of filamentous material known as the nucleoskeleton, is structured by type V intermediate filaments, which are mainly made up of lamins A and B [13,14]. Lamins play a wide range of roles contributing to nuclear structure and chromatin organization [15] and mutations in lamins especially in lamin A are associated with a large number of human diseases reminiscent of those found associated with the LINC complex [16]. Putative *Arabidopsis* homologues of intermediate filaments have been investigated by electron microscope studies [17] which revealed a meshwork of fibers underlying and connected to the NE and Nuclear Pore Complexes (NPCs), and by bioinformatics [18], genetics [19,20] and characterization of a lamina-like proteome [21]. The best candidates for lamin-like proteins in plants are the NUCLEAR MATRIX CONSTITUENT PROTEIN (NMCP) discovered in carrot and its *Arabidopsis* orthologues CRoWded Nuclei (CRWN) [19,20]. The four *Arabidopsis* CRWN*s* have long coiled-coil domains. Two of them (CRWN1 and CRWN4) are located at the nuclear periphery, while CRWN2 and CRWN3 are localized to the nucleoplasm [19,21]. Interaction of CRWNs with SUN1 and SUN2 has been demonstrated [22]. Like animal lamin mutants which show small, deformed nuclei, *crwn1* and *crwn4* mutants have small, more rounded nuclei [21,23], though this is not seen for *crwn2* or *crwn3*. Plants with a *crwn1* mutation combined with *crwn2, crwn3* or *crwn4* have even smaller nuclei [23].

A further component of the plant lamina is KAKU4, discovered in a mutant screen, where the mutants have smaller, spherical nuclei [24]. In *Arabidopsis*, KAKU4 interact with CRWN1 and CRWN4 and its overexpression results in NE overgrowth. Other novel components which are likely to play a role in the plant nucleoskeleton are the Nuclear Envelope Associated Protein (NEAP) family [25]. NEAPs comprise coiled-coil domains, a nuclear localization signal and a predicted transmembrane domain near the C-terminus. AtNEAPs interact with themselves and with both Cter- and mid-SUNs, and over expression relocates CRWN1 from the nuclear periphery to the nucleoplasm [25]. NEAPs and KAKU4 function in the lamina have yet to be fully explored.

## Impact of the plant nuclear periphery on nuclear features

### Nuclear shape and size

Nuclear morphology includes nuclear volume/size and nuclear shape and displays a large range of variation in plants. It is a complex trait as nuclear, cell and organ sizes are tightly regulated in a coordinated manner [26]. In animals, the nucleus is most often depicted as round or oval while its shape is variable [27], can be altered in some diseases [28] and during aging [26]. Plant nuclei are also polymorphic in size and shape as observed in different tissues [29,30]. Nuclear shape is thought to be stabilized by the connection of INM and ONM to one another at nuclear pores and to interactions at the INM between INM proteins, the nuclear lamina and chromatin and at the ONM by forces from the cytoskeleton all of which are likely mediated through the LINC complex. Many genes altering nuclear size and shape have been identified and most of them encode components of the microtubules [31], ONM [32,33], INM [6], NPC [34] and the lamina-like CRWN family referred to previously [23,24] and KAKU4 [24]. Surprisingly, most if not all these mutations induce smaller and rounded nuclei with the exception of mutants of GIP (γ-TuC Protein 3 (GCP3)-Interacting Proteins, involved in nucleating microtubules at the nuclear periphery [35,36] while in human cells, ghost shaped nuclei are observed [37].

Poulet et al [30] explored the role of components of the LINC complex, including KASH (*wifi: wip1 wip2 wip3 wit1 wit2* quintuple mutant) and SUN (*sun1 sun4 sun5* triple mutant) domain proteins in determining nuclear shape, size, heterochromatin organization, chromocentre position and activity of transcriptionally silent repetitive sequences. They observed altered nuclear shapes in three cell types investigated for SUN or KASH proteins and a nuclear lamina component (*crwn1 crwn2* double mutant). Mikulski et al [38] observed that the chromatin- and polycomb-associated component, PROLINE-TRYPTOPHANE-TRYPTOPHANEPROLINE INTERACTOR OF POLYCOMBS1 (PWO1), associates with CRWN1 in foci at the nuclear periphery and work together to maintain nuclear morphology as well as controlling expression of a group of target genes.

How might changes in the LINC complex and associated proteins as observed by Poulet et al [30] affect nuclear function? Two main hypotheses exist. The first hypothesis posits that changes in nuclear shape alter the rigidity of the nucleus; this could be beneficial for cells that need to squeeze through tight spaces, but deleterious to cells that are under mechanical duress. The second hypothesis proposes that changes in nuclear shape results in chromatin reorganization and thereby affects gene expression. It is important to note that these two hypotheses are not mutually exclusive [27].

### Nuclear movement

Plant nuclei are able to move in numerous cell types and circumstances, both as part of developmental processes or as a result of both biotic and abiotic stimulation [39]. Several cell models have been used to investigate nuclear movement in *Arabidopsis* such as root hair in which the nucleus migrates toward the tip as the root hair is growing [40]. KAKU1 a myosin XI–i homologue is involved in nuclear movement in root hairs. Such movements require the cytoskeleton especially actin filaments [32]. *wit1 wit2* mutant seedlings also impairs nuclear movement in root hair and Myosin XI–i was shown to interact with WIT1 and WIT2. Finally, WIT proteins are interacting with the KASH proteins WIP proteins anchored at the ONM [7,32,41]. This cascade of interaction nicely illustrates the connection between the nuclear envelope with the cytoskeleton and one of the key function of the LINC complex.

Another cell model is the pollen tube. In this case, during fertilization, the two sperm cell nuclei (SCN) and the vegetative nucleus (VN) migrate in the pollen tube to the unfertilized ovule. Zhou and Meier [42] demonstrated that WIT and WIP proteins are localized at the nuclear envelope of the VN and that they are essential for VN movement. Loss-of-function mutations in *WIT* and/or *WIP* gene families resulted in impaired VN movement that was no longer coupled with pollen tube tip growth. Zhou et al [33] went on to prove that the entire LINC complex was involved. Goto et al [43] recently show a role for the putative nuclear lamina protein KAKU4 in this process. KAKU4 is highly abundant in VNs and less so in SCNs. *kaku4* mutants show reversal in the order of migration of these two types of nuclei in elongating pollen tubes and reduced virility of the pollen.

### Chromatin structure and gene expression

Certain proteins present at the nuclear periphery participate in the regulation of gene activity by modifying the organization of chromatin. Two different aspects can be considered depending on whether broader chromatin domains or single gene transcription is considered.

Several studies have investigated the *A. thaliana* chromocentres which are heterochromatin domains enriched in silenced repeated sequences [44]. Chromocentres are of interest for this review as they are preferentially located at the nuclear periphery [30,45]. One of the most obvious impacts of the nuclear periphery on chromocentre organisation was observed with CRWN proteins. Fluorescent *in situ* Hybridization (FiSH) experiments clearly indicated that CRWNs are involved in cohesive forces within and between chromocentres as the double *crwn1 crwn2* mutant induces chromocentre fusion while more diffuse and decondensed chromocentres are observed in *crwn4* [23]. *crwn1* and *crwn4* single mutants were also studied by chromosome conformation capture (3C) and its whole genome application (Hi-C) [46–49]. *crwn1* and *crwn4* show an increased chromatin interactions between chromatin regions usually organised in different compartments and this is associated with an increased inter-chromosomal interactions. Finally, in a more general screen using 3D imaging investigating the effect of *crwn1, crwn4, kaku4, neap1* and *neap3* single mutants in *Arabidopsis*, Hu et al [49] suggested CRWN1 to be one of the key components maintaining specific chromatin domains at the nuclear periphery. Some effects were also recorded using the triple SUN mutant *sun1 sun4 sun5* in which chromocentres become more internal, being partially decondensed and leading to the derepression of some heterochromatic sequences [30].

As previously mentioned, CRWN1 interacts with PWO1, a component of the Polycomb-Group (PcG) associated factor. The PcG complex is a repressive complex and targeted genes are enriched in histone repressive marks such as H3K27me3. Interaction of PWO1 and CRWN1 in a transient expression assay revealed that this occurs to some extent near the nuclear periphery. Both proteins control expression of a similar set of target genes including those enriched in H3K27me3 [38,50]. Further studies show potential interaction of CRWN family members with transcription factors. CRWN1 interacts with NAC transcription factor NTL9 [51]. CRWN1 may also have the potential to directly interact with chromatin as supported by the recent ChIP-Seq analysis using a CRWN1:2 HA line performed by Hu et al [49]. These sequences were named Plant Lamina-Associated Domains (PLADs), and nicely overlap with chromatin domains linked (or next or associated) to the nuclear periphery previously identified by Restriction Enzyme (RE)-ChIP [52]. Target sequences are mostly silent chromatin domains enriched in repressive chromatin marks, with low transcription and include some transposable elements which are hypothesized to be anchor points for CRWN1 chromatin binding at the nuclear periphery.

As described above, AtNEAP, another component of the nucleoskeleton, interacts with AtbZIP18, a transcription factor containing a DNA-binding BRLZ domain, a leucine zipper allowing bZIP dimerization and an Ethylene-responsive element binding factor-associated Amphiphilic Repression (EAR) motif which is implicated in transcriptional inhibition through chromatin modification [53,54]. The EAR motif has been shown to recruit histone deacetylase 19 (AtHDA19) leading to gene repression [54]. AtbZIP18 contributes to pollen development in which it could act as a repressor, as single mutant *Atbzip18* mostly induces upregulation of pollen expressed genes [53].

All the interactions described above suggest some interactions between the nuclear periphery and chromatin. The pioneer experiments performed by the group of Chang Liu with CRWN1 strongly support the existence of specific chromatin organization at the nuclear periphery in plants [49,52] reminiscent of the Lamin Associated Domains (LADs) well-described in animal genome.

## Impact of the plant nuclear periphery on plant physiology

### Seed maturation and germination

Maturation of seed is an essential step for the survival of many plant species. This developmental period is critical to prepare the seed to survive to desiccation but to produce the young seedling for the next-generation. Together with various external signals such as light and temperature, phytohormones play a pivotal role in the regulation of seed germination. This regulation relies on the phytohormones abscisic acid (ABA) which promotes seed germination and gibberellin which inhibits seed germination by promoting seed maturation and dormancy. First evidence of a possible connection between the nucleoskeleton and the ABA pathway came from the investigation of the impact of nuclear size and chromatin organization on seed maturation and germination. ABA Insensitive 3 (ABI3) was shown to be required for the nuclear size reduction during seed maturation while CRWN1 and CRWN2 were required for the size increase during seed germination [55]. It is here interesting to note that one possible explanation of nuclear size regulation by CRWNs could be linked to their ability to induce membrane envelope overgrowth a phenomenon observed when CRWN are overexpressed in transient expression [24]. Membrane overgrowth is also observed when KAKU4 is over-expressed and this phenotype is even more pronounced when CRWN and KAKU4 are over-expressed together [24].

Further evidence of a connection between nuclear size and ABA pathway came from the investigation of the regulation of ABI5 degradation through the 26S proteasome pathway. ABI5 which is a key positive regulator in the ABA pathway is not only regulated by ABI3 but also by CRWN3 which is proposed to recruit ABI5 to specific nuclear body where it is degraded. This process mediated by the 26S proteasome pathway is dependent on the C-terminal of CRWN3 [56].

### Reproduction and meiosis

Meiosis is a specialized cell division required for plant sexual reproduction. Through a single S phase and two rounds of cell division, gametes are produced with half the chromosome number. During this, the ordered movement of chromosome is essential to achieve the pairing of homologous chromosomes. Chromosome movement is driven by forces generated in the cytoplasm conveyed by NE proteins to the ends of chromosomes (*i.e*. at telomeres). Chromosome attachment to the NE requires tubulin and actin [57] and involves the LINC complex components SUN1 and SUN2 [58,59]. Studying pollen mother cells (PMCs) in prophase I demonstrated that AtSUN1 and AtSUN2 localized to the NE [58], in a pattern resembling telomere location and connected by a thread structure like those formed in yeast meiosis [60]. An *Atsun1 Atsun2* double mutant shows impaired meiosis. Polarized telomere location to the NE in leptonema does not take place, prophase I is delayed, there is incomplete synapsis and unresolved interlocks, univalents are observed at metaphase I and missegregations at anaphase I that lead to the formation of aneuploid gametes [58]. One interesting hypothesis is that the bouquet formation (i.e. clustering of telomeres at the INM) with telomere pairing may promote homologous chromosome synapsis, an important step toward recombination events to generate crossovers. Finally, components of the nuclear periphery not only affect meiosis but also fertilization as they are key components in nuclear migration during pollen tube growth as already mentioned in the nuclear movement section [42,43].

### Organ development

The LINC complex is central to the processes of meiosis and mitosis which result in production of seeds and vegetative organs and therefore to the processes of cell and organ growth. The topic has been reviewed in detail recently [61]. Briefly, the behavior of the Cter-SUNs, SUN1 and SUN2 has been studied in mitotic division in synchronized tobacco cells [62]; in yeast and animals, they are known to be essential for centromere and centrosome anchorage and chromatin decondensation at the end of mitosis [63]. In *A. thaliana*, SUN1-YFP and SUN2-YFP fluorescence decreases and then increases in intensity at opposite sides of the NE as chromosomes condense. The NE then breaks down and is penetrated by spindle microtubules. SUN1 is then present in ER membranes around the mitotic spindle. As cells traverse anaphase, SUN1-YFP is present in tubule like structures around the segregated chromosomes. Both SUN1-YFP and SUN2-YFP are then located on decondensing chromatin facing the spindle poles before the membrane – containing the Cter-SUNs surround the chromatin. SUN1 and SUN2 aggregate first at the surface of chromatin facing the spindle pole, then at the periphery of the spindle, and finally facing the cell plate, a spatial organization also observed for NMCP1 and NMCP2 in *Apium* [64]. The new cell wall is now formed by expansion of a membrane structure known as the phragmoplast, within which the cell plate grows centripetally. Other NE associated components also accumulate at the phragmoplast, including the Nucleoporin Rae1, Ran, RanGAP, WITs, and WIPs [65,66].

### Response to stress

Plants are constantly facing changing environmental conditions such as biotic and abiotic stresses that can seriously affect their life cycle and productivity. In the past few years many reports have emerged on the contribution of the nuclear periphery to alter the plant immune system. As mentioned above, in *Arabidopsis* CRWN1 interacts with NAC WITH TRANSMEMBRANE MOTIF1-LIKE9 (NTL9). This interaction enhances NTL9 binding at PATHOGENESIS-RELATED1 (*PR1*) gene repressing *PR1* transcription, a key gene in plant defense mediated by the salicylic acid (SA) pathway. Therefore, CRWN1 is expected to be part of a repressive mechanism keeping silenced *PR1* [51]. This elegant analysis also delivered more mechanistic features of CRWN1 action. If CRWN1 transcription is induced by SA or upon bacterial infection, it is rapidly degraded by a proteasome-dependent pathway. Thus, reducing CRWN1 abundance contributes to PR1 induction by transcription factors such as NONEXPRESSOR OF PR GENES1 (NPR1). NPR1 is a major transcriptional activator of PR1 which is imported through the NPC during biotic response. NPC and nucleoskeleton thus have complementary functions, CRWN maintaining the repression of PR genes through a possible alteration of chromatin marks [67] until signaling molecules such as NPR1 transcription factor release this repression. Further analyses of transcription at a whole genome scale highlighted the induction of genes from the SA pathway in *crwn* mutants [51,67]. In an attempt to better understand the complex mutant phenotype observed in *crwn* mutants *i.e* small plant phenotype, small nuclei, induction of SA, *crwn* mutants were combined with mutations affecting the SA pathway such as *NPR1* or *ISOCHORISMATE SYNTHASE1* (*ICS1/SID2*). *crwn1 crwn2 npr1* [51] and *crwn1 crwn2 sid2* [67] compromised the small plant phenotype of *crwn1 crwn2* meaning that activation of SA response was indeed strongly contributing to the small plant phenotype of the *crwn* mutants. However, the small nuclei phenotype remained, suggesting that CRWN function regulation of nuclear morphology and SA-response can be disconnected [67]. Finally, recent evidence suggests that CRWN1 protein not only affect SA signaling but also jasmonic acid (JA) signaling in *Arabidopsis* [68] opening avenues to explore its impact on a more widespread pathogen response including not only bacteria but also fungal diseases.

Returning to the variation of nuclear size observed during seed maturation and germination, Van Zanten et al [55] suggested that the decreased nuclear size could represent a universal mechanism in acquisition of desiccation tolerance. However the possible dehydration tolerance of *CRWN* mutants which display a reduced nuclear size has not been tested. The authors measured nuclear size of leaves of the desiccation-tolerant resurrection plant *Craterostigma plantagineum* in normal and dehydrated conditions and found a reduced nuclear size in stressed condition suggesting that alteration of nuclear size can also occur in leaves. In *A. thaliana*, SUN3 might play an important role in stress tolerance by modulating UPR signaling, possibly via ABA-independent pathways. The wild-type and the mutant plants were found to be compromised in their tolerance to dehydration as indicated by increased transpirational water loss [69]. Finally, CRWN-family proteins were recently shown to protect against Reactive Oxygen Species (ROS) accumulation and against DNA damage [70].

All together, these reports suggest that the plant nuclear periphery acts as a sensor to external stresses especially in biotic stress response.

## LINC complex and associated components in crop species

### Conservation in monocot and dicot cultivated species

Decades of breeding have improved yield and the ability for plants to better respond to various stresses. Plant breeding is a constantly evolving strategy and from the green revolution in the 1960–70’s to the current genomic selection era, major progress has been made to enhance plant growth and genetic pools. Understanding plant physiology and its adaptation to changing environments is timely and knowledge transfer between model and cultivated plants has contributed and still offers good opportunities to identify new ways of improvement to reach this major challenge for the future. Because genetics by its own cannot explain all the phenotypic plasticity of plants, epigenetics is often cited for its ‘strong potential for crop breeding’ [71,72].

**Figure 1.**
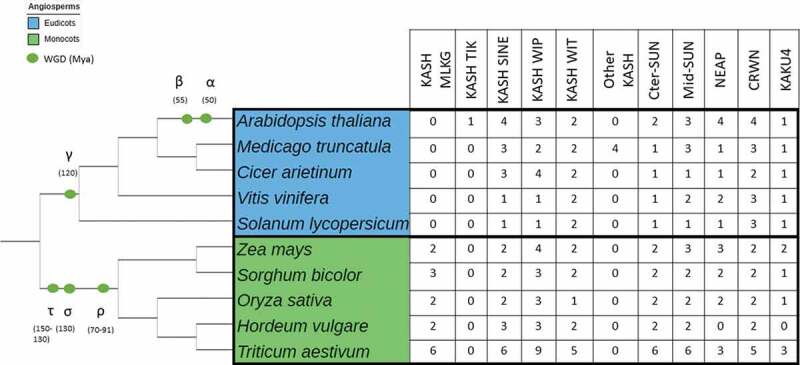
Number of Paralogues of LINC complex and associated components in monocot and dicot lineages

The nuclear periphery, as one layer of this complex regulation, described in the previous sections, actively participates in the transmission of external signals through the nuclear envelope to alter chromatin organization (*i.e*. epigenetics). However, investigation of plant LINC complexes and associated proteins in crop species remains sparse although many proteins can be identified by Blastp analysis or by exploring databases (Table 1, Figure 1).

**Table 1.** LINC complex and associated components in monocot and dicot lineages

Species	Nuclear Envelope									Nuclear periphery	
	LINC complex								INM	Nucleoskeleton	
	KASH proteins (ONM)						SUN proteins (INM)		NEAP	CRWN/NMCP	KAKU4
	LINC KASH grass-specific	KASH TIK	KASH SINE	KASH WIP	KASH WIT	Other KASH	Cter-SUN	Mid-SUN			
*A. thaliana* (TAIR+ Poulet et al., 2016)		**AtTIK** (AT5G44920)	**AtSINE1** (AT1G54385) **AtSINE2** (AT3G03970) **AtSINE3** (AT3G06600) **AtSINE4** (AT4G24950)	**AtWIP1** (AT4G26455) **AtWIP2** (AT5G56210) **AtWIP3** (AT3G13360)	**AtWIT1** (AT5G11390) **AtWIT2** (AT1G68910)		**AtSUN1** (AT5G04990) **AtSUN2** (AT3G10730)	**AtSUN3** (AT1G22882) **AtSUN4** (AT1G71360) **AtSUN5** (AT4G23950)	**AtNEAP1** (AT3G05830) **AtNEAP2** (AT5G26770) **AtNEAP3** (AT1G09470) **AtNEAP4** (AT1G09483)	**AtCRWN1** (AT1G67230) **AtCRWN2** (AT1G13220) **AtCRWN3** (AT1G68790) **AtCRWN4** (AT5G65770)	**AtKAKU4** (AT4G31430)
*M. truncatula* (Newman-Griffis et al., 2019 + PLAZA Dicots 4.5)			**MtSINE1** (Mt6g032885) **MtSINE5a** (Mt2g033900) **MtSINE5b** (Mt5g054260)	**MtWIP1a** (Mt8g070940) **MtWIP1b** (Mt3g009740)	**MtWIT1** (Mt7g017690) **MtWIT2** (Mt3g086660)	**MtKASH1** (Mt3g099060) **MtKASH4** (Mt4g036225) **MtKASH5** (Mt6g016290) **MtKASH6** (Mt1g052620)	**MtSUN1** (Mt8g043510)	**MtSUN4-a** (Mt3g006320) 450/2e-150 **MtSUN4-b** (Mt1g069420) 436/3e-143 **MtSUN4-c** (Mt4g030960) 421/3e-140	**MtNEAP2** (Mt1g484830) 444/1e-155	**MtCRWN1** (Mt7g018610) 859/0.0 **MtCRWN2** (Mt6g015285) 588/0.0 **MtCRWN4** (Mt4g097580) 252/2e-67	**MtKAKU4** (Mt3g108780) *197/1e-53*
*C. arietinum* (PLAZA Dicots 4.5)			**CaSINE1-a** (Ca_13211.g) 534/0.0 **CaSINE1-b** (Ca_15536.g) 517/2e-177 **CaSINE2** (Ca_15305.g) 62.4/2e-08	**CaWIP1-a** (Ca_01362.g) 87.4/5e-18 **CaWIP1-b** (Ca_20564.g) 85.1/4e-17 **CaWIP1-c** (Ca_17833.g) 83.6/4e-17 **CaWIP2-b** (Ca_20519.g) 174/1e-46	**CaWIT1** (Ca_16511.g) 369/4e-117 **CaWIT2** (Ca_15964.g) 421/1e-139		**CaCter-SUN** (Ca_15702.g) 441/1e-150	**CaMid-SUN3/CaSUN1** (Ca_21229.g) 427/3e-142	**CaNEAP2** (Ca_03601.g) 373/4e-128	**CaCRWN2** (Ca_10456.g) 644/0.0 **CaCRWN4** (Ca_09369.g) 240/1e-63	**CaKAKU4** (Ca_01833.g) 182/2e-48
*V. vinifera* (Poulet et al., 2016 + PLAZA Dicots 4.5)			**VvSINE1** (GSVIVG01033391001)	**VvWIP** (GSVIVG01014846001)	**VvWIT1** (GSVIVG01036512001) 517/4e-174 **VvWIT2** (GSVIVG01010127001) 479/4e-160		**VvCter-SUN** (GSVIVG01001935001)	**VvSUN3/4** (GSVIVG01037194001) **VvSUN5** (GSVIVG01019558001)	**VvNEAP2** (GSVIVG01031318001) **VvNEAP3** (GSVIVG01028212001)	**VvCRWN1** (GSVIVG01031076001) **VvCRWN3** (GSVIVG01011972001) **VvCRWN4** (GSVIVG01007428001)	**VvKAKU4** (GSVIVG01035528001)
*S. lycopersicum* (Poulet et al., 2016 + PLAZA Dicots 4.5)			**SlSINE1/2** (Solyc03g062700.2)	**SlWIP** (Solyc07g066170.2)	**SlWIT1** (Solyc02g065660.2) 383/1e-122 **SlWIT2** (Solyc05g010590.2) 395/6e-128		**SlCter-SUN** (Solyc01g096780.2)	**SlSUN5** (Solyc08g082540.2)	**SlNEAP** (Solyc01g101130.2)	**SlCRWN1-a** (Solyc03g045050.2) **SlCRWN1-b** (Solyc02g089800.2) **SlCRWN4** Solyc02g091960.2	**SlKAKU4** (Solyc08g006480.2)
*Z. mays* (Gumber et al., 2019)	**MLKG1** (Zm00001d002723) **MLKG2** (Zm00001d038539)		**MLKS1** (Zm00001d031134) **MLKS2** (Zm00001d052955)	**MLKP1** (Zm00001d003334) **MLKP2** (Zm00001d025667) **MLKP3** (Zm00001d005997) **MLKP4** (Zm00001d020879)	**MLKT1** (Zm00001d039272) **MLKT2** (Zm00001d010047)		**ZmSUN1** (Zm00001d015450) **ZmSUN2** (Zm00001d040331)	**ZmSUN3** (Zm00001d042643) **ZmSUN4** (Zm00001d012240) **ZmSUN5** (Zm00001d011277)	**MNEAP1** (Zm00001d010918) **MNEAP2** (Zm00001d024756) **MNEAP3** (Zm00001d002875)	**NCH1** (Zm00001d051600) **NCH2** (Zm00001d043335)	**MKAKU41** (Zm00001d026487) **MKAKU42** (Zm00001d002012)
*S. bicolor* (PLAZA Monocots 4.5)	**SbMLKG1** (Sb06g024760/Sobic.006G172400) 295/2e-99 **SbMLKG2-a** (Sb09g023990/Sobic.009G179500) 282/2e-95 **SbMLKG2-b** (Sobic.004G352400) 87.4/3e-19		**SbSINE1-a** (Sb05g022560/ Sobic.005G164900) 421/3e-139 **SbSINE1-b** (Sb08g022270/ Sobic.008G179600) 230/3e-66	**SbWIP1-a** (Sb02g027920/ Sobic.002G242200) 93.6/1e-18 **SbWIP1-b** (Sb07g028540/ Sobic.007G218900) 77.4/2e-13 **SbWIP3** (Sb06g019690/ Sobic.006G115466) 57.8/3e-07	**SbWIT1-a** (Sb09g008110/ Sobic.009G088000) 296/5e-89 **SbWIT1-b** (Sb03g000580/Sobic.003G004600) 261/1e-75		**SbSUN1** (Sb04g005160/Sobic.004G061900) 306/5e-98 **SbSUN2** (Sb03g010590/Sobic.003G125332) 262/7e-81	**SbSUN4-a** (Sb03g041510/ Sobic.003G374700) 399/2e-130 **SbSUN4-b** (Sb03g026980/ Sobic.003G208900) 327/1e-102	**SbNEAP2-a** (Sb09g002860/ Sobic.009G033800) 344/2e-116 **SbNEAP2-b** (Sb03g011900/Sobic.003G139700) 239/3e-75	**Sb CRWN1** (Sb04g030240/ Sobic.004G264300) 446/3e-136 **SbCRWN4** (Sobic.003G308200) 494/8e-157	**SbKAKU4** (Sb06g031220/ Sobic.006G246900) 65.9/1e-09
*O. sativa* (Poulet et al., 2016 + PLAZA Monocots 4.5)	**OsMLKG1** (Os04g0554300/LOC_Os04g46790) 120/7e-32 **OsMLKG2** (Os02g0824600/LOC_Os02g57850) 136/3e-38		**OsSINE1** (Os11g0580000/LOC_Os11g37100) **OsSINE2** (Os12g0624800/LOC_Os12g42960)	**OsWIP1-a** (Os04g0471300/ LOC_Os04g39540) **OsWIP1-b** (Os08g0497900/ LOC_Os08g38890) 82.0/6e-15 **OsWIP1-c** (Os09g0481300/ LOC_Os09g30350) 77.8/1e-13	**OsWIT1** (Os05g0241000/ LOC_Os05g15140) 314/1e-95		**OsSUN1/SAD1** (Os05g0270200/LOC_Os05g18770) **OsSUN2** (Os01g0267600/ LOC_Os01g16220)	**OsSUN3** (Os01g0876400/LOC_Os01g65520) **OsSUN5** (Os01g0599900/LOC_Os01g41600)	**OsNEAP1** (Os05g0135900/LOC_Os05g04530) **OsNEAP2** (Os01g0292700/ LOC_Os01g18840) 81.6/4e-16	**OsCRWN1** (Os02g0710102/LOC_Os02g48010) **OsCRWN4** (Os01g0767000/LOC_Os01g56140)	**OsKAKU4** (Os04g0655600/LOC_Os04g56140)
*H. vulgare* (PLAZA Monocots 4.5)	**HvMLKG1** (HORVU2Hr1G097210) 103/9e-25 **HvMLKG2** (HORVU2Hr1G110520) 94.4/2e-22		**HvSINE1-a** (HORVU6Hr1G028190) 435/4e-146 **HvSINE1-b** (HORVU5Hr1G006220) 206/1e-56 **HvSINE1-c** (HORVU5Hr1G006040) 87.8/5e-17	**HvWIP2-a** (HORVU5Hr1G071910) 83.6/2e-15 **HvWIP2-b** (HORVU2Hr1G081800) 58.9/8e-08 **HvWIP3** (HORVU7Hr1G048940) 69.3/8e-11	**HvWIT1-b** (HORVU7Hr1G007940) 288/2e-85 **HvWIT1-a** (HORVU3Hr1G057330) 284/2e-85		**HvSUN1-a** (HORVU1Hr1G019590) 275/7e-86 **HvSUN1-b** (HORVU3Hr1G037960) 264/2e-81	**HvSUN3** (HORVU3Hr1G081880) 383/8e-123 **HvSUN5** (HORVU3Hr1G052360) 313/3e-98		**HvCRWN1** (HORVU6Hr1G069560) 464/5e-143 **HvCRWN4** (HORVU3Hr1G074480) 263/6e-74	
*T. aestivum* (PLAZA Monocots 4.5)	**TaMLKG1** TraesCS2B02G430400 88.6/4e-19 **TaMLKG2-a** TraesCS6D02G218400 155/6e-45 **TaMLKG2-b** TraesCS6A02G235700 137/9e-38 **TaMLKG2-c** TraesCS6B02G264300 129/5e-35 **TaMLKG2-d** TraesCS6D02G386000 74.3/1e-14 **TaMLKG2-e** TraesCS6B02G444900 58.2/1e-08		**TaSINE1-a** TraesCS6A02G144100 444/7e-148 **TaSINE1-b** TraesCS6B02G172500 442/2e-147 **TaSINE1-c** TraesCS6D02G133300 441/9e-147 **TaSINE1-d** TraesCS5B02G019900 207/2e-57 **TaSINE1-e** TraesCS5D02G028500 201/2e-55 **TaSINE1-f** TraesCS5A02G022900 201/3e-55	**TaWIP2-a** TraesCS5A02G257000 84.7/1e-15 **TaWIP2-b** TraesCS5B02G256500 82/7e-15 **TaWIP2-c** TraesCS5D02G265600 81.3/1e-14 **TaWIP2-d** TraesCS2A02G331000 65.1/2e-09 **TaWIP2-e** TraesCS2D02G332000 63.2/7e-09 **TaWIP2-f** TraesCS2B02G351300 57/3e-07 **TaWIP3-a** TraesCS7B02G135800 86.7/2e-16 **TaWIP3-b** TraesCS7A02G240200 64.7/2e-09 **TaWIP3-c** TraesCS7D02G238200 55.5/1e-06	**TaWIT1-a** TraesCS3A02G158400 291/6e-87 **TaWIT1-b** TraesCSU02G064900 287/2e-85 **TaWIT1-c** TraesCS7D02G038700 282/2e-83 **TaWIT1-d** TraesCS7A02G042800 281/5e-83 **TaWIT1-e** TraesCS3D02G166100 263/2e-76		**TaCter-SUN1-a** TraesCS1B02G108700 277/9e-87 **TaCter-SUN1-b** TraesCS1D02G090900 276/2e-86 **TaCter-SUN1-c** TraesCS1A02G109200 276/4e-86 **TaCter-SUN1-d** TraesCS3A02G184300 268/2e-83 **TaCter-SUN1-e** TraesCS3B02G214100 267/4e-83 **TaCter-SUN1-f** TraesCS3D02G188400 266/1e-82	**TaMid-SUN3-a** TraesCS3D02G379800 407/3e-132 **TaMid-SUN3-b** TraesCS3A02G389900 405/7e-131 **TaMid-SUN3-c** TraesCS3B02G418900 401/8e-130 **TaMid-SUN3-d** TraesCS3D02G216900 320/4e-100 **TaMid-SUN3-e** TraesCS3A02G214200 318/5e-99 **TaMid-SUN5** TraesCS3B02G244900 316/2e-99	**TaNEAP2-a** TraesCS1D02G081500 325/4e-109 **TaNEAP2-b** TraesCS1A02G079800 323/5e-108 **TaNEAP2-c** TraesCS1B02G097800 300/7e-99	**TaCRWN1-a** TraesCS6D02G256200 472/6e-146 **TaCRWN1-b** TraesCS6B02G303400 469/9e-145 **TaCRWN4-a** TraesCS3B02G337300 516/3e-166 **TaCRWN4-b** TraesCS3A02G299200 516/8e-165 **TaCRWN4-c** TraesCS3D02G302500 486/8e-154	**TaKAKU4-a** TraesCS2A02G503200 91.7/9e-18 **TaKAKU4-b** TraesCS2B02G531200 89.7/4e-17 **TaKAKU4-c** TraesCS2D02G504000 88.2/1e-16

### *Similarities and differences between* Arabidopsis *and crop species*

Significant differences are observed among plant genomes regarding gene copy number which is not surprising considering the plasticity of plant genome size [73]. Plant genomes were subjected to several rounds of Whole Genome Duplications (WGD) such as the ρ-σ-τ WGD and the α-β-γ WGD series respectively in monocot and dicot lineages [74]. WGDs partially explain the high variability in paralogue numbers. For instance *Arabidopsis* would theoretically contain up to 3 (γ) x 2 (β) x 2 (α) (*i.e*. 12 expected paralogues) paralogues of the ancestral genes found in the basal angiosperm *Amborella trichopoda* if these had not been eliminated during evolution while species such as *Solanum lypersicum* or *Vitis vinifera* will have been subjected only to the γ triplication (*i.e*. 3 expected paralogues) [75]. Thus, one can expect that the number of copies would be lower in these species as observed in Table 1 and Figure 1. Polyploid genomes such as wheat (allohexaploid species) contains not only paralogues but also three homeologues. This also explains the higher number of copies identified in wheat *versus* barley, rice and maize that are all diploid species. Finally it is also important to note that *Arabidopsis* with its rather small genome of about 150Mb contains a limited number of transposable elements (TEs, ~10% of the genome) mainly located at pericentromeric regions, while maize (3Gb genome) and wheat (~16Gb genome) have high number of TEs (up to 80% of their genome) dispersed along their chromosomes [73]. Regarding these genome features, *Arabidopsis* may then appear as an outlier among plant species. But to go further in these differences before going back to the observed functional similarities, genome size is not the only difference between *Arabidopsis* and crop genomes as chromosomes may adopt distinct organization in the nucleus. Some like yeast or wheat display a Rabl-like organization with telomeres and centromeres at opposite sides of the nucleus while others such as rice, maize, Sorghum and *Arabidopsis* do not [76–80]. Telomeres are also at various positions, being close to heterochromatin regions in tomato, dispersed around the nuclear periphery in wheat and rice and tightly associated with the nucleolus in *Arabidopsis* [79].

### Crop species as model to study the impact of LINC complex and associated components in plant traits

Despite the clear differences that should also be kept in mind, *Arabidopsis*, maize and rice chromosome telomeres all form a bouquet structure and interact with the nuclear periphery during meiotic prophase 1 [58,81,82]. In all three cases, the SUN family is involved. Following the simple concept of looking for functional similarities, reports about LINC complex and associated components have been collected from a small number of cereals and legume species as referred in Table 1 and Figure 1.

#### Cereals species

Rice (*Oriza sativa*) was the first plant species in which a SUN (SAD) protein was described, localized to NE and ER [83]. Recently, Shah et al [84] have studied the rice SUN domain proteins in depth and observe that all the rice SUN genes respond to abiotic stress (drought, salinity, temperature). Zhang et al [82,85] further studied the behavior of rice SUN domain proteins in meiosis. Using an *Ossun1, Ossun2* double mutant, Zhang et al [85] observed severe defects in telomere clustering, homologous pairing and crossover formation, indicating an essential role for both Cter-SUNs. Further analysis of the mutants, in a line in which topoisomerase initiated homologous recombination was disrupted, suggested that OsSUN1 and OsSUN2 are not completely redundant in function as the *Ossun1* single mutant had a normal phenotype but meiosis was disrupted in the *Ossun2* mutant. Finally, Yang et al [86] have recently described the interaction between OsNMCP1 and *Oriza sativa* SWITCH/SUCROSE NONFERMENTING (SWI/SNF) silencing complex. This interaction occurs between OsNMCP1 and OsSWI3C under drought stress conditions leads to the release of OsSWI3C from the SWI/SNF gene silencing complex, thus changing chromatin accessibility in the genes related to root growth and drought resistance.

Maize (*Zea mays*) SUN domain proteins were classified in CCSD (canonical C-terminal SUN domain) and PM3-type (plant-prevalent Mid-SUN 3) [5]. Five genes for SUN domain proteins have been identified [5]. ZmSUN1 and ZmSUN2 are Cter-SUNs, while ZmSUNs 3–5 are mid-SUNs (Table 1). Murphy and Bass [59] studied the role of SUN domain proteins in maize meiosis. Maize forms a bouquet in zygotene. SUN2 is concentrated into a belt-like structure at the nuclear periphery which reorganizes to a half-belt in zygotene and returns to a full belt in pachytene. The half belt is associated with the cluster of telomeres in the bouquet and coincides with synapsis of homologous chromosomes. The half belt location of the SUN domain proteins is disrupted in the meiosis-specific mutants, *desynaptic* (*dy1), asynaptic1* (*as1*), and *divergent spindle1* (*dv1*). Based on the maize model, Bass proposed that first telomeres move to the NE, cluster together during the prezygotene-zygotene phase and connect to the microtubule system to move the nucleus within the cell and allow proper meiotic recombination. For these reasons, the LINC complex, which applies forces needed to tether telomeres to the nuclear envelope, has a major contribution in the meiotic process [81].

Further evidence for the functional components of the LINC complex, CRWN, KAKU4 and NEAPs in maize have been obtained in studies led by the Bass laboratory [12]. Using a combination of bioinformatics and cell biology, 22 genes have been identified and characterized that interact with SUN2 including the novel graminaceous KASH family (MLKG 1–2) (Table 1). MLKG1 interacts with SUN and is localized to the NE and ER actin network. Interaction with ZmSUN2 *in vivo* has been shown for MLKP2 and MLKG1; however, unlike most KASH domain proteins where the KASH domain is essential for NE localization, KASH deletion constructs remain associated with the nuclear periphery, suggesting other interactions that associate them with NE. More functional validation are now expected in maize thanks to this pioneer study which will strongly benefit the previous *Arabidopsis* studies. To our knowledge, there were no reports in other important species such as barley, wheat and sorghum.

#### Legume species

*Medicago truncatula* is a model legume used to explore the processes of nodulation. Zhou et al [9] and Newman-Griffis et al [87] explored its LINC complex components using a combination of bioinformatics, protein protein interaction and mutant analysis. They identified one *Medicago* SUN domain protein (MtSUN), and putative homologs of the *Arabidopsis* KASH proteins WIP1 (MtWIP1a, MtWIP1b) SINE1 (MtSINE1) and a further SINE family member, named SINE5 by Zhou et al (2014), MtSINE5a and MtSINE5b (Table 1). They also described the WIT1 and WIT2 homologues MtWIT1 and MtWIT2 and described a role for LINC complex components in root hair responses to environment that are involved in initiation of the symbiotic formation of the nodule. *Medicago* also contains a number of other KASH genes not attributable to known families designated MtKASH1, MtKASH4, MtKASH5 and MtKASH6 (Table 1, Figure 1). The additional complexity of the legume LINC family may be associated with its role in nodulation; however, homologues of these additional KASH genes were not detected in *Cicer arietinum*, the Chickpea, the second main cultivated grain legume (Table 1, Figure 1). SUN proteins were also investigated in Chickpea. Only one Cter-SUN (CaCterSUN) and one mid-SUN (CaSUN1) was detected in Chickpea while one homologue of CRWN1-3 (CaCRWN2) and one homologue of CRWN4 (CaCRWN4) was found (Table 1, Figure 1). One gene for KAKU4 (CaKAKU4) is also present. CaSUN1 is similar to AtSUN3 and was identified by mass spectrometry as one out of the 280 genes downregulated under dehydration [69].

## Concluding remarks

**Figure 2.**
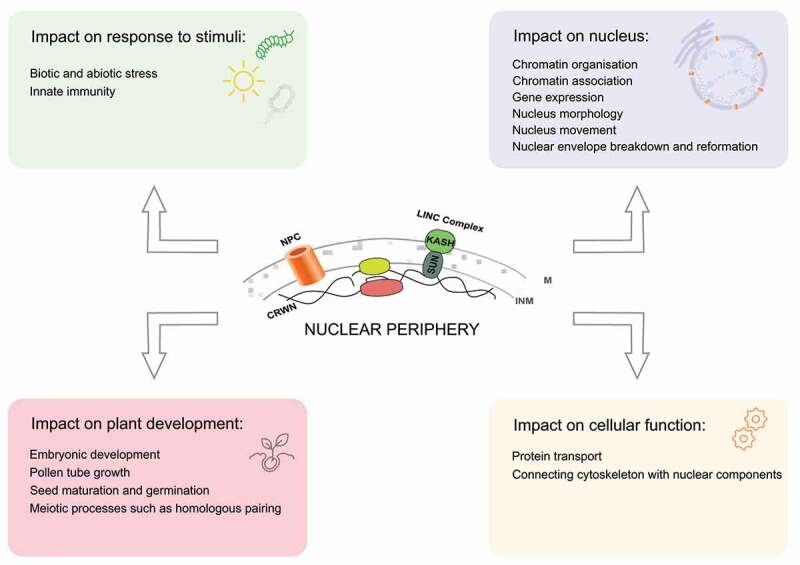
Schematic representation summarizing key functions of nuclear periphery components in plants

**Figure 3.**
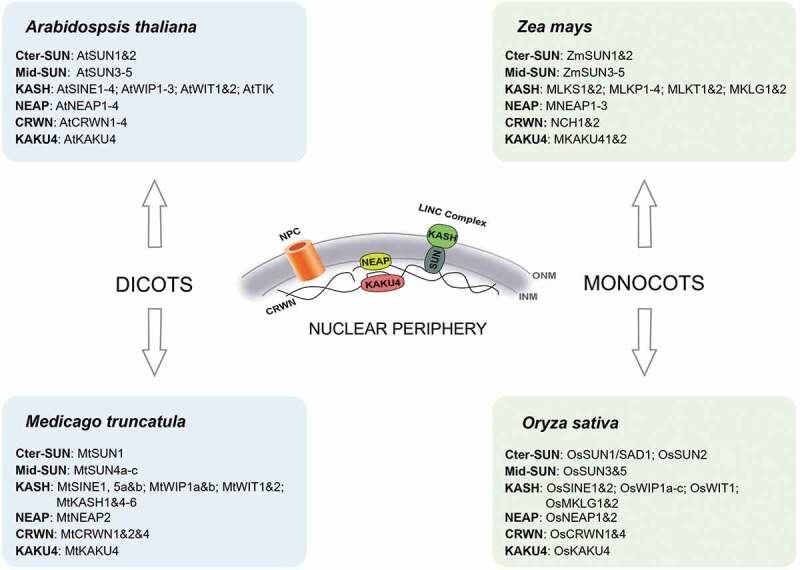
Schematic comparison of nuclear periphery components for monocot and dicot crops and the model species *Arabidopsis thaliana.*

This review focuses on advances in knowledge of the physical structure of the nucleus and the mechanisms by which nuclear dynamics might in the future be manipulated to enhance productivity and response to environmental factors. Increasingly, crop scientists are looking beyond classical genetics and genomics to consider epigenetic effects- modification to gene expression without changing the genetic code. Recent advances show that some of these epigenetic determinants rely on the structure of the nucleus, the position and movement of genes as well as of the nucleus itself which are all-important in controlling the activity of the genome. As described in the first part of this review, the LINC complex and associated components regulate key functions in various aspects of the plant cycle (Figure 2). Most of them are conserved in a wide panel of plant species while some are Brassicaceae (KASH TIK) or monocot (KASH MLKG) specific (Figure 3). We have no doubt that more reports from cultivated species will come out in the coming years. However, application in plant improvement will have to face the pleiotropic effects of these genes and their impact on plant growth. Yield can be severely impaired in some of the mutants as observed in the constitutive activation of SA signaling pathway in *crwn* mutants inducing spontaneous expression of chlorotic lesions in the absence of pathogens due to hypersensitive response (HR) [67]. This class of mutation was well described by maize geneticists almost 40 years ago as lesion-mimic mutants [88] and was considered as a model system to understand plant cell death in plants. Will this inconvenience prevent any application in agronomy? One can also expect that during evolution, each paralogue even each homeologue will diverged from its original ancestral homologue and acquired specific expression profiles and functions. As some of these genes are key players in nuclear/cell/organ size, meiosis, seed germination and stress responses, we propose there should be targeted research aimed at their functional characterization in crop species. Crops benefit from many genetic resources including TILLING populations, germplasm collections genome editing or advanced crossing populations [89] that will strongly benefit to the functional study of the nuclear periphery components.

A simple phylogenetic tree according to the Tree of Life [90] using 10 plant species is proposed on the left side of the figure. The proposed tree starts with angiosperms divergence (~170 Mya) and include the main whole Genome Duplications (WGD) relative to the dicot (blue) and monocot (green) lineages indicated as green circles on tree branches. Timing estimation of WGD events expressed in Million years (Mya) are indicated in between brackets [74,75,91]. τ is anterior to ρ and close to the origin of monocots is not completely resolved [91]. Number of paralogues is given on the right for each species for 11 gene families.

Species used in this table: *A. thaliana: Arabidopsis thaliana, M. truncatula: Medicago truncatula, C. arietinum: Cicer arietinum, V. vinifera: Vitis vinifera, S. lycopersicum: Solanum lycopersicum, Z. mays: Zea mays, S. bicolor: Sorghum bicolor, O. sativa: Oryza sativa ssp. Japonica, H. vulgare: Hordeum vulgare, T. aestivum: Triticum aestivum*

This table was produced from data found in the literature as well as in the databases available on PLAZA [92] using the Monocots PLAZA 4.5 for *T. aestivum, H. vulgare, O. sativa* and *S. bicolor* and Dicots PLAZA 4.5 for *S. lycopersicum, V. vinifera, C. arietinum* and *M. truncatula*. The protein sequences of *A. thaliana* or *Z. mays* (for MLKG proteins) were used as query sequence to perform Blastp on PLAZA with a cut off of 1e-05. The score in bits and the e-value of results of Blastp are written after the name of corresponding gene. The proteins noted in color are those found in the literature while the others are those found from databases. The different colors represent the different publications used in the table: blue: Poulet et al., 2016 [1], orange: Newman-Griffis et al., 2019 [87] and green: Gumber et al., 2019 [12].
